# Time to Sleep?—A Review of the Impact of the COVID-19 Pandemic on Sleep and Mental Health

**DOI:** 10.3390/ijerph19063497

**Published:** 2022-03-16

**Authors:** Vlad Sever Neculicioiu, Ioana Alina Colosi, Carmen Costache, Alexandra Sevastre-Berghian, Simona Clichici

**Affiliations:** 1 Department of Microbiology, “Iuliu Hatieganu” University of Medicine and Pharmacy, 400012 Cluj-Napoca, Romania; icolosi@umfcluj.ro (I.A.C.); anca.costache@umfcluj.ro (C.C.); 2 Department of Physiology, “Iuliu Hatieganu” University of Medicine and Pharmacy, 400012 Cluj-Napoca, Romania; berghian.alexandra@umfcluj.ro (A.S.-B.); sclichici@umfcluj.ro (S.C.)

**Keywords:** sleep, sleep deprivation, sleep duration, sleep quality, COVID-19, mental health

## Abstract

Sleep is intrinsically tied to mental and overall health. Short sleep duration accompanies the modern lifestyle, possibly reaching epidemic proportions. The pandemic and subsequent lockdowns determined a fundamental shift in the modern lifestyle and had profound effects on sleep and mental health. This paper aims to provide an overview of the relationship between sleep, mental health and COVID-19. Contrasting outcomes on sleep health have been highlighted by most reports during the pandemic in the general population. Consequently, while longer sleep durations have been reported, this change was accompanied by decreases in sleep quality and altered sleep timing. Furthermore, an increased impact of sleep deficiencies and mental health burden was generally reported in health care workers as compared with the adult general population. Although not among the most frequent symptoms during the acute or persistent phase, an increased prevalence of sleep deficiencies has been reported in patients with acute and long COVID. The importance of sleep in immune regulation is well known. Consequently, sleep deficiencies may influence multiple aspects of COVID-19, such as the risk, severity, and prognosis of the infection and even vaccine response.

## 1. Introduction

Sleep is highly conserved in the animal kingdom, essential to the maintenance of homeostasis in our body. Sleep is tied to the physiology of the human body, and as such, sleep requirements vary significantly for different age groups. The NSF (National Sleep Foundation) recommends 14–17 h for new-borns, 9–11 h for school aged children, 8–10 h for teenagers, 7–9 h for young adults and adults and 7–8 h of sleep for older adults [1].

Sleep plays a fundamental role in both overall and mental health. Sleep and mental health are profoundly linked at a neurobiological level [2]. Both decreased sleep duration or quality can adversely impact mental health [3,4], and conversely, most mental health disorders present with objective or subjective alterations in sleep [2,5]. The causal relation between altered sleep and decreased mental health is evidenced by the fact that improvements in sleep translate to improvements in mental health such as depression, anxiety, rumination and stress [6].

**Figure 1:**
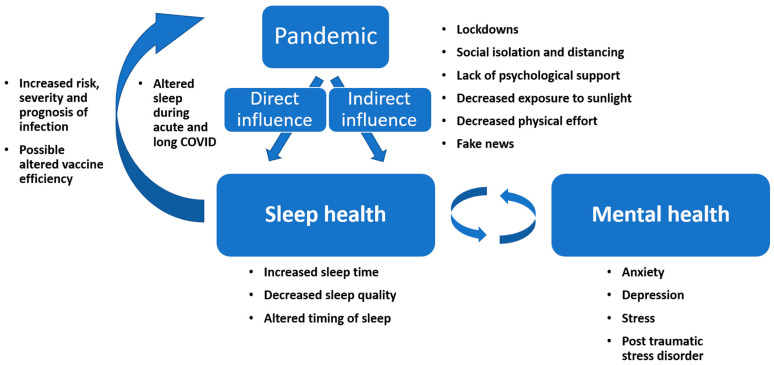
The bidirectional relationship between COVID-19 and sleep/mental health.

This paper aims to provide an overview of the relationship between sleep/mental health and COVID-19. In order to achieve this goal, multiple aspects of this relationship were examined: the epidemiology of sleep deprivation and sleep deficiencies before and during the COVID-19 pandemic, the effect of sleep deficiencies on COVID-19 parameters (risk, severity and vaccine efficiency), sleep changes during acute and long COVID and the effect of the pandemic on mental health. Regarding sleep health, although the main focus has been on reduced sleep duration, frequent elements of sleep quality, circadian misalignment, sleep disorders or general sleep disturbances and deficiencies have also been included. A visual summary of the bidirectional relationship between COVID-19 and sleep/mental health can be found in Figure 1.

Multiple terms can be used to describe the reduction in sleep time or to at least include a reduction in sleep duration: sleep deprivation, sleep loss, sleep deprivation/insufficient sleep syndrome [7,8], sleep insufficiency [9], or broader terms such as sleep deficiency [10], sleep wake disfunction [11]. Although there are subtle differences between these terms, occasionally they are used interchangeably in the literature. Beyond examining specific elements of sleep disruption, a relatively new term can be used to obtain a global view of sleep—namely, sleep health. Sleep health integrates multiple elements of sleep, such as duration, timing, regularity, efficiency and satisfaction [12].

In order to promote health, sleep needs to be characterised by “adequate duration, good quality, appropriate timing and regularity and the absence of sleep disturbances or disorders” [13]. Although both sleep quantity and quality are fundamental for the restorative effects of sleep, a more important role of sleep quality has been suggested in assessing sleep health [14]. Ultimately, sleep quality and quantity are interconnected, representing different fundamental aspects of healthy sleep [15].

Risk factors that affect sleep health frequently overlap, simultaneously affecting multiple sleep parameters. Therefore, sleep insufficiency and disruption can be caused by a wide range of factors, including behavioural/lifestyle (voluntary sleep loss, sleep loss due to social or work obligations) and psychosocial and medical conditions (sleep disorders and other medical conditions) [9,16]. Globalisation and the advent of a 24/7 society both regarding work and leisure activities are other factors that can have detrimental effects on sleep [17].

Alterations in sleep duration, quality or any other dimension of sleep health have profound detrimental effects ranging from daytime sleepiness, cognitive impairment and altered mood to cardiovascular disease, obesity, type 2 diabetes and immunosuppression [9,18].

The current pandemic was declared a short time after the emergence of a novel coronavirus in late December 2019. The virus was subsequently named SARS-CoV-2 [19] and the infection caused by it COVID-19. Subsequent lockdowns, social distancing and isolation, determined widespread unique lifestyle changes with a significant impact on sleep and mental health.

## 2. Materials and Methods

As presented before, the terminology surrounding sleep deficiencies is sometimes used interchangeably in the literature. In addition, the literature concerning sleep/mental health and COVID-19 is often characterised by a marked heterogeneity both regarding study protocols and published results. Due to these facts, we structured this paper as a narrative review in order to extend the scope of the review and better include multiple relevant aspects of sleep deficiencies and mental health that were altered by the pandemic.

We performed multiple broad PubMed searches between 10 September and 15 October 2021 by including the following keywords and MeSH terms: “sleep”, “sleep deprivation”, “sleep insufficiency”, “insufficient sleep”, “epidemiology”, “COVID-19”, “COVID”, “coronavirus”, “mental health”. For the three major subsections of this review (epidemiology of sleep deprivation and deficiencies before and during the pandemic; the relationship between mental health, sleep deficiencies and COVID-19) the following simplified search protocols were employed: (sleep OR sleep deprivation OR sleep insufficiency OR insufficient sleep OR sleep deficiencies) AND epidemiology; (sleep OR sleep deprivation OR sleep insufficiency OR insufficient sleep OR sleep deficiencies) AND (COVID-19 OR COVID OR SARS-CoV-2 OR coronavirus); (sleep OR sleep deprivation OR sleep insufficiency OR insufficient sleep OR sleep deficiencies) AND (mental health OR anxiety OR depression OR PTSD OR post-traumatic OR stress) AND (COVID-19 OR COVID OR SARS-CoV-2 OR coronavirus). Only high-quality studies were included, prioritising systematic reviews, meta-analyses and large sample studies. For sections where sufficient data were not yet available, we also included studies with reduced sample sizes. In addition, relevant websites such as the WHO (World Health Organisation), CDC (Centers for Disease Control and Prevention), NIHR (National Institute for Health Research), NHLBI (National Heart, Lung, and Blood Institute) were consulted. References of included studies were screened, and the subsequent papers were added to the review if relevant to the subject.

## 3. Epidemiology of Sleep Deprivation before the Pandemic

### 3.1. Epidemiology of Sleep Deprivation before the Pandemic

The widespread occurrence of insufficient sleep has prompted some authors to consider it an epidemic with a wide range of public health implications [20]. Most available evidence seems to point towards decreases in both sleep time and quality associated with the modern lifestyle, but despite this fact, an agreement on the existence of a sleep loss epidemic has not yet been reached.

Although a global phenomenon, significant differences in the prevalence of sleep deprivation can be seen between different countries, ages and timeframes. Among adults, multiple estimates from the 1990s and 2000s place the prevalence of short sleep between 7.5% and 28.3% [21,22,23,24,25,26]. With the exception of some reports [27,28], more recent evidence points out that approximately one-third of the adult population is sleep deprived: 29.2–35.2% [26,29,30,31]. If objective sleep data are taken into consideration, the prevalence of short sleep has been reported to reach even 47% [28].

Sleep deprivation also represents a significant issue in the younger segment of the population, with a prevalence between 11.7% and 72.7% being reported [28,31,32,33,34,35,36,37,38]. With the exception of some reports [36,37], the prevalence of sleep deprivation in this population group can be estimated between one-third and two-thirds. Overall, short sleep seems to be more prevalent on school nights as compared with free nights or weekends [31,33,36,38]. This observation was further confirmed by a recent meta-analysis, showing that a peak in the weekday–weekend difference of time spent in bed can be observed in teenagers [28]. Insomnia [39] and other sleep disturbances [40] are also frequent in this age group. One of the direct outcomes of reduced sleep duration is sleepiness. As evidenced by multiple studies [18,28] sleepiness seems to be more frequent in adolescents as compared with the general population. In partial accordance with the presented data, Chattu et al. [18] reported that over half of middle school (57.8%) and high school students (72.7%) experience sleep deprivation.

Among the older segment of the population the prevalence of sleep deprivation has been estimated between 11.9% and 53.9% [31,41,42,43,44,45,46,47,48], with most reports indicating that one-fourth of this population segment experiences short sleep durations. Overall, sleep complaints are common in the ageing population and are frequently determined by a multitude of factors ranging from the physiologic effects of ageing to comorbidities and medication use. Chronic sleep problems (40–70%) and insomnia symptoms (~50%) are commonly reported, carrying a significant health impact in this age group, as reviewed by Miner at al [49]. Furthermore, self-reported decreased sleep quality is also prevalent in low income settings among older adults, with as many as 40% of individuals reporting severe sleep problems [50].

In contrast with the presented data, a recent meta-analysis highlighted the fact that although 24.5% of individuals of all age groups experience short sleep, only a reduced percentage (5.8%) sleep less than the acceptable sleep duration. According to the authors, reduced sleep quality and insomnia symptoms were more prevalent as compared with reduced sleep duration [28].

A detailed account of the presented data can be found in Table 1 for adults and in Table 2 and Table 3 for the younger and ageing segment of the population, respectively.

### 3.2. Is Sleep Deprivation an Epidemic?

Even though sleep deprivation is common in the modern world, several arguments can be brought against its extent to epidemic proportions.

A comparison drawn between modern sleep habits and those of pre-industrialised societies revealed relatively similar sleep durations of 6–7 h. Interestingly, this comparison highlighted some important sleep characteristics only present in hunter–gatherers: a seasonal difference in sleep duration between summer and winter, awakening that takes place before sunrise, maximal sunlight exposure in the morning and limited napping, among others [51].

Another argument for the limited extent of sleep deprivation is provided by conflicting results presented by studies that examined sleep trends over the last decades. As such, while multiple studies found no significant decreases in sleep time [52,53], others found conflicting results [54,55] or country-dependent changes with both increases, decreases and stationary trends in sleep habits [56,57].

As opposed to the adult population, trends in sleep duration for children and adolescents seem to indicate a decrease of ~1 h over the last century, with country-dependent changes [58].

## 4. Epidemiology of Sleep Deprivation during the Pandemic

Sleep deficiencies have been observed with increased frequency during the pandemic in HCW (health care workers), the general population and patients with COVID. Collectively, sleep deficiencies observed in the context of the pandemic have been termed ‘coronasomnia’ in the media and by some authors [59,60,61].

### 4.1. Direct Comparisons Drawn before and during the Pandemic

In order to evaluate the effect of the pandemic on sleep, multiple studies have drawn a direct comparison between data collected before and during the course of the pandemic. Overall, conflicting results have been reported. While some studies reported a relatively constant sleep time [62], multiple others reported an increase in overall sleep time [63,64,65,66], with most studies reporting a decrease in sleep quality [62,63,64,66].

Changes in sleep habits reported by adults from the USA revealed similar sleep durations and decreased sleep quality as compared with before the pandemic. Additionally, significant increases in difficulties falling asleep, staying asleep and feeling unrested were also reported. Interestingly, despite the fact that sleep time remained largely unchanged, increases in the extremes of both short (34% to 40.7%) and long sleepers (4.2% to 7.4%) have been observed [62]. Self-reported data from a sample comprised of participants from six different countries revealed an overall modest increase in sleep duration among the general population (0.31 h, 95% CI 0.24–0.39) but not in HCW during lockdown, and a decrease in sleep quality was reported by approximately one-third of individuals [63]. Results from the ECLB COVID-19 international online survey revealed a decrease in sleep quality coupled with a modest increase in sleep duration and reduced physical activity in adults. In this instance, sleep quality was evaluated through the PSQI (Pittsburgh Sleep Quality Index): 5.32 ± 3.23 as compared with 4.37 ± 2.71 before lockdown. Overall, 39% of individuals reported poor sleep before lockdown, as opposed to 52% during lockdown [64].

Multiple positive changes in sleep health have been observed among adolescents during the pandemic, including an increase in self-reported sleep duration and delay in sleep timing. As such, a decrease in individuals not obtaining the recommended hours of sleep has been highlighted (<8 h of sleep; 20% before the pandemic, 9.5% during the pandemic). As noted by the authors, the delayed school time could have benefited the physiologically delayed bed and wake times in this age group, thus significantly decreasing sleep debt and social jet lag [65]. In accordance with this point of view, delayed school start times have been shown to be an effective method for promoting sleep health [67].

As in the case of adults, significant decreases in sleep quality and physical activity have been observed among the ageing population during the pandemic. Despite the fact that the mean sleep duration marginally increased during lockdown (6.8 h to 6.96 h), both values were slightly below the recommended sleep duration. Global poor sleep quality (PSQI) increased from pre- to during lockdown (48% to 57% of individuals), with significant increases in sleep latency and decreases in sleep efficiency among others. Noteworthy in this population group, both sleep quality and physical activity were found to be predictors of decreased mental health pre- and during the pandemic [66].

### 4.2. Data from Systematic Reviews and Meta-Analyses during the Pandemic

Multiple systematic reviews and meta-analyses aiming to ascertain the prevalence of sleep deficiencies during the pandemic were published recently [68,69,70,71,72,73,74]. Most of the aforementioned studies used broad definitions for sleep deficiencies, such as sleep problems [68,72], sleep disturbance [69,70,73] and sleep disorders [71], while one meta-review evaluated the prevalence of insomnia [74]. Most of the mentioned studies included subgroup analyses according to the population segment, and some included analyses based on the questionnaire used to determine sleep problems.

Depending on the diagnostic method used, the prevalence of sleep deficiencies among the adult, younger segment of the population and HCW were reported between 18% and 32.3% [68,72], 54% [73] and between 31% and 45.1% [68,70,71,72], respectively.

Taking multiple population groups into consideration (general population, HCW, COVID-19 patients), over one-third (35.7%) of individuals presented sleep deficiencies [68]. A similar prevalence of insomnia was also reported among the general population and HCW (32.34%) [74].

Although estimative, an increased prevalence of sleep deficiencies seems to be present in HCW as compared with the general population [68,70,71,72]. A detailed comparison of sleep deficiencies in the adult general population and HCW is presented in Table 4.

Xia et al. [70] reported an insightful subgroup analysis according to the line of work and subsequent exposure to COVID-19 of HCW in China. Sleep deficiencies were more common in infected (97%) and frontline HCW (57.4%) as compared with non-frontline HCW (40%). Consequently, the overall sleep health of HCW in Wuhan was decreased as compared with the sample mean (as evidenced by an increased PSQI value of 10.87).

As highlighted in a recent systematic review, due to marked heterogeneity of the data, large ranges of prevalence of sleep disturbances can be observed both in the case of the general population (17.65–81%) and HCW (18.4–84.7%) [69].

Among the younger segment of the population, over half of children (54%) exhibited sleep disturbances during home confinement, and almost half (49%) were not meeting the recommended sleep duration for their age [73].

As a global phenomenon, decreases in sleep health (both quantity, quality and timing) have also been observed in multiple other large population-based studies, of which most employed online questionnaires. The reported effects on sleep range from altered timing of sleep [75,76,77,78], decreases in sleep quality [75,76,77,78,79,80,81], shortened sleep duration [79], prolonged sleep duration or increased time spent in bed [76,77,78] and increased prevalence of insomnia or insomnia-related symptoms [79,82,83].

### 4.3. Objective Sleep Data during the Pandemic

A few recent studies investigated objective sleep data during the pandemic. Wrist actigraphy and self-reported sleep data revealed delayed bed and wake times, an increase in sleep duration and a general decrease in sleep quality as compared with the period before the pandemic (PSQI before pandemic 4.59 ± 2.86 and PSQI during pandemic 5.04 ± 3.02) [84]. Furthermore, data collected from smartphones and wearable devices showed similar results, with a shift in bed and wake time and increases in sleep duration [85,86].

## 5. Effects of Sleep Deficiencies on Risk, Severity of COVID-19 and Vaccine Efficiency

As reviewed by Besedovsky et al. [87], sleep is paramount for the homeostasis of the immune system, and as such, altered sleep can influence susceptibility to infections, infection outcome and immune response to vaccination.

Multiple large-scale population studies presented links between altered sleep and the risk of developing subsequent infection. Short sleep duration, a diagnosed sleep disorder and self-reported complaints about sleep quality were found to increase the likelihood of a respiratory infection [88], and both reduced and prolonged sleep durations were associated with an increased risk of pneumonia [89].

During the pandemic, HCW were exposed to increased workloads, both during the day and during night shifts. Working during the night shift has been previously shown to decrease antiviral immunity (through decreases in TNF-α) and to influence a possible inflammatory response to infection (through mistimed release of IL-6) [90]. In accordance with these findings, two recent studies among HCW presented multiple risk factors for COVID-19 infection: higher BMI (body mass index), night shift work, decreased sleep time, sleep problems and burnout [91,92]. A higher BMI and night shift work have been associated with an increased risk of infection, while self-reported longer sleep time has been found to be protective against infection with SARS-CoV-2 [91]. Daily burnout was associated with increased risk, duration and severity of infection [92].

A recent meta-analysis examined multiple risk factors for COVID-19 susceptibility, severity of infection and subsequent death from infection. Pre-existing sleep disturbances and sleep deficiencies determined by the primary infection were associated with illness severity (pooled OR 1.62, 95%CI 1.36–1.94 and pooled OR 12.21, 95%CI 3.81–39.18). In addition, pre-existing mood disorders were associated with susceptibility, severity and death from COVID infection [93]. Furthermore, self-reported reduced sleep time prior to the COVID-19 diagnosis was also associated with risk of developing severe infection and with infection prognosis [94]. Similarly, Zhang et al. [95] reported an association between decreased sleep quality (PSQI and RCSQ—Richards–Campbell Sleep Questionnaire) and slow recovery from lymphopenia, increased risk of disease severity through higher risk of ICU (intensive care unit) admission and longer hospital stay in a small sample of patients hospitalised with COVID-19.

Previous experience with sleep deprivation in the context of vaccination has been shown to negatively influence multiple vaccination parameters and possibly interfere with vaccine efficiency in the case of vaccines for seasonal influenza, hepatitis A and B and 2009 H1N1 viruses. Therefore, it has been suggested that sleep quantity or quality may play a role in the efficacy of a COVID-19 vaccine [96]. At the time of writing, to our knowledge, no studies have been yet published addressing the relationship between sleep deprivation and vaccine efficiency against COVID-19. Future studies will likely address this question [97].

## 6. Quantitative and Qualitative Alterations of Sleep in Acute and Long COVID Patients

The importance of sleep for immune regulation is well known by now, but relatively few studies exist in the literature describing the effects of infections on sleep duration and quality.

Endotoxin, respiratory tract and generalised infections have been shown to influence sleep in different ways. Endotoxin may exhibit a dose-dependent effect on sleep, with lower doses increasing SWS (short-wave sleep) [98] and higher doses disrupting sleep [98,99]. While sleep time has been shown to be decreased during acute rhinovirus infections [100], another recent paper reported a prolonged sleep duration during ILI (influenza-like illness) and ARI (acute respiratory infection), along with alterations in sleep quality [101]. Furthermore, generalised infections such as HIV have also been shown to alter multiple sleep parameters, including a reduction in sleep duration [102].

Overall, changes in both sleep quantity and quality are common in infectious and non-infectious diseases, with a wide range of observed effects on sleep ranging from enhancing, reducing, disrupting or misplacing sleep [87]. A dose-dependent effect of immune activation on sleep parameters is suggested by data from animal models, ranging from sleep enhancement to sleep disruption: modest immune activation—increased NREM (non-rapid eye movement sleep) sleep with a possible restorative effect on immune homeostasis; pronounced immune activation (infection)—increased NREM and decreased REM (rapid eye movement sleep) sleep, changes that may support the host’s defence mechanisms; extreme immune activation (severe infection)—disruption of NREM and REM sleep, which determine sleep fragmentation, nonrestorative sleep and daytime sleepiness [87].

Sleep deficiencies have been described both during the acute COVID-19 infection and as persistent symptoms after the initial infection in COVID survivors. Long COVID has been defined in a recent NICE (National Institute for Health and Care Excellence) guideline as a syndrome of persistent signs and symptoms after the initial acute infection [103].

### 6.1. Sleep Deficiencies during Acute COVID-19

Although not among the most frequently described symptoms of acute COVID-19, an increased prevalence of sleep deficiencies has been reported by multiple recent meta-analyses ranging from 14.9% to 74.8% [68,72,104,105,106]. In partial accordance with the presented data, the range of prevalence of sleep deficiencies was determined to be 33.3–84.7% in a recent systematic review [69].

Despite the fact that only a relatively reduced percentage of patients will require hospitalisation during acute COVID-19 infection, significant importance must be placed on sleep deficiencies in relation to hospitalisation. It is well known at this point that both sleep time [11,107] and quality [11,107,108] are affected by hospitalisation in both adult [11,107,108] and paediatric patients [11]. Sleep-disturbing elements can be grouped as extrinsic (noise, irregular exposure to lights, bright lights alerted bedtime routine, clinical interventions, etc.) and intrinsic (primary illness, anxiety, depression, posttraumatic stress, physical pain, etc.) factors, as reviewed by Morse et al. [11]. Sleep disturbances during hospital stay can determine significant negative consequences, such as impaired recovery, prolonged hospitalisation and reduced self-reported well-being. Of note, one study examined the self-reported sleep quality in hospitalised patients with infection, revealing that 47.8% patients experienced unsound sleep [108]. Consequently, a high occurrence of sleep deficiencies and decreased mood has been reported early in the course of the pandemic in hospitalised patients treated in the isolation ward [109,110] and ICU [111] for COVID-19. Interestingly, the positive effect of muscle relaxation and psychological intervention on both sleep quality and anxiety/depression symptoms highlights the relationship between sleep and mental health in the context of COVID-19 [109,110,111]. An increased prevalence of insomnia and impact on mental health have also been highlighted in a small sample study of COVID-19 isolation ward patients [112]. These findings were confirmed by another study, underlining the decreased sleep quality in hospitalised COVID-19 patients through PSQI (mean 9.3 ± SD 4.6). Interestingly, an association between self-reported COVID-19 symptom severity and sleep quality/mental distress has been highlighted [113].

At the time of writing of the present report, limited evidence is available regarding objective sleep data in acute COVID-19 patients. In a small sample study, four patients showed sleep alterations measured by wrist actigraphy and PSQI during post-acute rehabilitation. Despite the small sample size, the authors observed that patients with increased severity of respiratory symptoms and prolonged ICU stay exhibited lower sleep quality (lower sleep efficiency and higher sleep fragmentation index) as compared with patients with milder symptoms [114].

At this point, the evidence presented in the literature is relatively scarce or frequently resulted from small sample sizes. Based on previous evidence regarding sleep in the context of infections and the available data, we believe that it is safe to assume that multiple sleep parameters have the potential to be altered during acute COVID-19 infection in both hospitalised and at-home patients.

### 6.2. Sleep Deficiencies during Long COVID

Evidence from previous SARS outbreaks confirmed the existence of persistent symptoms after the initial infection and classified them as a post-SARS syndrome. The syndrome was similar to fibromyalgia, with a wide range of symptoms such as chronic fatigue, decreased mental health and sleep disturbances. Sleep difficulties were common in SARS survivors at different timepoints after the initial infection (3 months—47%; 6 months—50%; 12 months—44%) [115]. Sleep disturbances such as nonrestorative sleep and EEG (electroencephalogram) anomalies were also reported among HCW at ~18 months follow-up after the initial infection [116].

Multiple elements regarding long COVID are unknown at the moment. Although a consensus has not yet been reached, multiple classifications and groupings of symptoms have been described, including a grouping of symptoms into three distinct categories: related to post-viral chronic fatigue, related to post-critical-illness syndrome and related to PTSD (posttraumatic stress disorder) [117].

The persistence of at least one symptom after ‘Long COVID’ has been described at multiple time points after the initial infection. A detailed account of the persistence of COVID-19 symptoms after the initial infection can be found in Table 5. In partial agreement with these data, a recent NIHR (National Institute for Health Research) review estimated the following prevalence of symptom persistence: in previously hospitalised patients 50–89% at 2 months; in non-hospitalised patients 20–30% at 1 month and at least 10% 3 months later [117].

According to the majority of the available data, fatigue seems to be the most commonly encountered symptom during ‘Long COVID’ [118,121,122,123,124,125,126]. Nevertheless, a high prevalence of insomnia or sleeping difficulties has been also described by multiple studies. A detailed account of the prevalence of insomnia or sleeping difficulties is presented in Table 6.

Due to the marked heterogeneity of the results and the use of different case definitions and different study populations, conclusions regarding the persistence of sleep difficulties or insomnia are difficult to draw. To our knowledge, the longest follow-up time after the acute infection was 12 months [122,124]. Although an overall trend in the presented data is not obvious, Huang et al. [124] reported a decrease in sleep disturbances from 6 to 12 months follow-up in patients previously hospitalised for acute COVID-19— from 27% to 17% of patients, respectively.

The persistence of sleep disturbances in the aforementioned studies, the data reported at 12 months follow-up [122,124] and the decrease in sleep disturbances over time [124] are in accordance with previous published papers in the literature. Sleep disturbances (both objective and self-reported) after hospital discharge have been observed in elderly patients [128] and are well known in discharged patients after critical illness [129,130]. Sleep disturbances in discharged patients after critical illness show an improvement over time but can persist for up to 12 months after discharge [129,130].

Similar findings were also presented in two studies that evaluated self-reported sleep disturbances in HCW. Compared with before the initial infection, overall sleep quality and multiple sleep parameters such as latency, duration, efficiency, use of sleep medication and daytime dysfunction were found to be significantly altered in HCW at two months after the initial COVID-19 infection [131]. Similarly, Gaber et al. [132] observed that almost half of HCW exhibited at least one symptom several months after the initial infection, with similar rates of sleep (49%) and mood (44%) disturbances. Additionally, HCW were less likely to take sick leave or seek medical advice.

The beforementioned studies described self-reported sleep disturbances, evaluated through questionnaires and interviews [120,121,122,123,124,125,126,127,131,132], and only one study reported PSQI values [131].

The prevalence of sleep deficiencies in patients with long COVID was reported in multiple recent systematic reviews and one meta-analysis between 29.4% and 47%: sleep disorders or insomnia 29.4% (median, IQR 24.4–33%) [133]; pooled prevalence of sleep disturbances 47% (95%CI 7–89) [134]; range of prevalence 29.5–40% [69].

Factors that lead to the appearance and persistence of symptoms after the initial COVID-19 infection are not well understood at this moment, but as noted by some authors, the likelihood of symptoms seems to increase with age [122,123]. The influence of the acute infection on symptom load and severity during long COVID is not well understood. Consequently, conflicting data are available in the literature. While one study highlighted an association between symptom load during the acute phase and symptoms at follow-up [119], several others seem to point out a lack of connection between the severity of the initial infection and more severe complications [122,125].

To our knowledge, limited evidence exists in the literature regarding objective measurement of sleep parameters after the initial COVID-19 infection. Three recent studies evaluated sleep parameters through PSG (polysomnography) at 1 month, 4–6 weeks and 4 months after the initial infection. In a case report, Goyal et al. [135] reported increased REM density and alpha intrusion in NREM/REM sleep at one month after the initial infection. Heidbreder et al. [136] reported an increased presence of RWA (REM sleep without atonia) in 36% of patients from a small sample (*n* = 11). In another study, 67 COVID ICU survivors with moderate to severe ARDS (acute respiratory distress syndrome) underwent PSG after 4–6 weeks from the initial infection. A high prevalence of moderate to severe OSA (obstructive sleep apnoea) of 73% was observed. As noted by the authors, untreated OSA could lead to cardiac complications and persistent symptoms such as fatigability and insomnia, thus partially explaining the increased prevalence of these symptoms in COVID-19 survivors [137].

As noted in a recent NIHR review [117] and by multiple authors [133,134], the marked heterogeneity of published studies prevents the drawing of conclusions and generalisations at this stage. Overall, a decreased quality of life and high impact of persistent symptoms including sleep deficiencies can be observed in COVID-19 survivors.

## 7. Mental Health during the Pandemic

An increased impact on mental health problems has been highlighted by multiple meta-analyses and systematic reviews in HCW [71,74,138], the general population [74,138,139] and acute [105,106,139] and surviving COVID patients [133,134]. Anxiety, depression, stress and PTSD symptoms appear to be the most prevalent mental health problems presented in the literature.

### 7.1. Mental Health Impact in the General Population and HCW

An increased impact on mental health has been observed both in HCW and the general population during the pandemic. Taking both groups into consideration, about 1 in 3 individuals experienced decreased mental health symptoms (psychological distress 28.25%; stress 36%; anxiety 27.77%; depression 26.93%; PTSD 19.58%) [74]. Overall, the available data seem to point to an increased burden of mental health symptoms in HCW as compared with the general population. Contrary to the previous statement, a recent meta-review highlighted a similar prevalence of anxiety and depression between HCW and the general population [74]. A more detailed comparison of the mental health impact of the pandemic between the general population and HCW is presented in Table 7.

### 7.2. Mental Health Impact in Acute and Surviving COVID Patients

Patients with acute COVID presented increased psychological symptoms, mainly regarding anxiety and depression. The prevalence of anxiety symptoms was reported between 15.9% and 47%: 15.9% [105], 39.6% [139], 47% [106]. Similarly, the prevalence of depression symptoms has been reported as 23% [105] and 45% [106].

An increased prevalence of mental health complaints has also been reported after the initial COVID infection. In surviving COVID patients, the pooled prevalence of anxiety/depression and decreased mental health or PTSD symptoms were reported as 37.5% (95% CI 19–58%) and 14.5% (95% CI 4–29%), respectively [134]. In a systematic review, the median frequency percentages for depression and anxiety in COVID survivors were reported as 14.9% (IQR 11–18%) and 22.1% (IQR 10–29.6%) [133].

### 7.3. Mental Health Impact on Pre-Existing Mental Health Conditions and Vulnerable Groups

Relatively limited evidence is available on the impact of a pandemic on pre-existing mental health conditions. Evidence from previous pandemics and from data published early during the COVID-19 pandemic suggests an increased effect of the pandemic on persons with pre-existing mental health conditions. Compared with controls, people with pre-existing mental health conditions experience increased psychiatric, anxiety and depression symptoms [140]. Gobbi et al. [141] reported the worsening of self-reported mental health symptoms concerning general psychological disturbance, PTSD and depression, in over half of participants in an online survey. Similar results were reviewed by Murphy et al. [142], highlighting the exacerbation of symptoms in pre-existing mental health conditions. In contrast with these findings, Nam et al. [143] reported mixed results regarding the mental health impact in vulnerable groups such as people with chronic disease, pregnant women and people with pre-existing mental health conditions. Interestingly, the older segment of the population experienced lower levels of stress, anxiety and depression symptoms.

The impact of the pandemic and social isolation is also evident in children and young people. Loneliness determined by the pandemic landscape has been associated with anxiety and depression in the young segment of the population with pre-existing mental health conditions [144].

## 8. Mental Health and Other Factors That Can Influence Sleep during the Pandemic

It is well known in the literature that mental health and sleep are bidirectionally linked. As such, the high prevalence of mental health symptoms determined by the pandemic can at least partially explain the increased prevalence of sleep disorders. A negative feedback loop can thus be established with sleep problems further exacerbating the already present mental health symptoms.

Multiple other factors may also partially explain the increased impact of sleep deficiencies seen during the pandemic. Among HCW, increased workloads may further exacerbate the stress generated by the current pandemic. Being a frontline COVID-19 worker, being a nurse, a lower education level and less work experience, the lack of sufficient protective equipment along with worries regarding the pandemic, being infected and lack of psychological support are all factors that have been shown to negatively influence sleep. The pandemic and subsequent lockdowns have the potential to generate or exacerbate existent circadian disturbances through reduced exposure to sunlight during the day, limited physical activity and significant changes in work or school schedule. Despite the mentioned points, positive elements to sleep health have been observed during the pandemic. These changes were determined by reduced social jetlag, reduced sleep restriction and an overall reduced sleep debt [69]. The increased sleep duration reported by multiple studies was frequently coupled with a reduction in sleep quality. This fact was probably determined by the psychological impact of the pandemic. Although any illness is accompanied by psychological stressors, the current pandemic presented new psychological challenges, such as those derived from the transmission of the virus, uncertainty, over- or misinformation, isolation from loved ones, etc., as noted by Yang et al. [111].

In order to explain the increased prevalence of sleep disorders among COVID-19 survivors, some authors proposed a multifactorial model, taking into consideration the primary infection, social isolation and decreased physical activity [122], while others proposed the role of decreased mental health (anxiety, depression, PTSD, stress) [134]. Etiologically, decreased mental health and psychological symptoms could be explained by direct (acute infection and an aberrant immune response ranging from hyperactivation to autoimmunity) and indirect factors (loneliness, reduced social contact, incomplete recovery, loss of employment) [124]. In addition to the proposed hypotheses, the exposure of hospitalised patients to forced early awakenings, sedative medication and the environment of the isolation ward can further decrease the quality of sleep [114].

Another possible explanation for increased sleep disturbances in acute and surviving COVID patients could be determined by the relationship between pain and sleep. Pain and sleep are known to be bidirectionally linked: sleep can be disrupted by pain and alterations in sleep duration or quality can lower the pain threshold [145].

## 9. Limitations in the Interpretation and Comparison of Literature Data

Due to the nature of our review and due to a non-systematic search of the literature, key elements regarding the relationship between COVID-19 and sleep/mental health could have been overlooked. Furthermore, multiple aspects of the current literature surrounding COVID-19 and sleep/mental health hinder comparisons and generalisations. As such, definitive conclusions are difficult to draw.

### 9.1. Heterogeneity Regarding Sleep Health Terminology

One limitation in the comparison between studies regarding sleep and COVID-19 is represented by the heterogeneity of the terms that are used surrounding sleep, with an important proportion of studies using broad terms such as sleep problems, sleep disturbances or sleep deficiencies.

### 9.2. Subjective and Objective Sleep Data

Another limitation in the interpretation of population-wide sleep studies is determined by contrasting results obtained through self-reported data (questionnaires, sleep diaries, etc.) and objectively measured (PSG, actigraphy) sleep durations. It is well known that self-reported sleep duration is often biased through overestimation in population-wide studies [146,147]. The extent of the overestimation has been reported as ~20–30 min [147,148,149], reaching even ~60 min in some instances [147,150].

Several sleep disorders are known to induce a subjective–objective mismatch in sleep data, such as insomnia and obstructive sleep apnoea. Regarding insomnia, it is well known that individuals with insomnia frequently underestimate their subjective sleep time [151,152]. Misperception of sleep time may also be present in obstructive sleep apnoea patients, to a certain extent [152,153]. While a subjective–objective mismatch in sleep perception is common in insomnia patients, extreme cases are considered to belong to a separate clinical entity—paradoxical insomnia (previously sleep state misperception) [8,154]. Many unknowns remain regarding the altered perception of sleep in patients with insomnia. Some evidence seems to point out the role of psychological factors (such as cognitive activity before sleep and mood) in the misperception of sleep, thus partially explaining it [155]. Additionally, depression and anxiety symptoms seem to be more frequent in individuals with paradoxical insomnia [156]. However, recent evidence seems to indicate that psychopathology does not play a significant role in sleep misperception [157]. In contrast with psychological factors, sleep fragmentation seems to play an insignificant role in the subjective–objective mismatch of sleep in insomnia or OSA patients [152,158].

In addition to the described disorders, mood at awakening also plays a key role in the discrepancy between objective and subjective sleep data. As such, a negative association between positive mood and the subjective–objective mismatch has been described in older adults [159].

Therefore, studies with self-reported sleep data must be interpreted cautiously, with the possible assumption of overestimated values regarding sleep time in the general population and an underestimation in individuals suffering from insomnia.

Nevertheless, subjective sleep data especially regarding sleep quality play an important role in the global evaluation of sleep health, providing additional useful information as compared with objective measurements [160].

### 9.3. Heterogeneity Regarding Used Sleep Questionnaires

The use of multiple questionnaires (ranging from validated questionnaires such as PSQI, RCSQ, SRSS (Sleep State Self-Rating Scale) to components of validated questionnaires or simple questions about sleep time or quality is another factor that hinders comparisons between studies. As evidenced by one meta-analysis, the prevalence of sleep disturbances varied from 19% to 55%, depending on the diagnostic method used (PSQI vs. custom questionnaire) [106].

### 9.4. Diagnosed vs. Total COVID-19 Cases

The reported prevalence of sleep deficiencies during the acute infection and long COVID only took data from confirmed cases into consideration. Due to limitations in testing capabilities and multiple other factors, undiagnosed COVID-19 cases far outnumber the confirmed cases. During the early stages of the pandemic, it has been proposed that as many as 98–99% [161], or 1 in 3 ranging to 1 in 1104 [162] acute COVID-19 cases went undiagnosed and unreported. Even with expected improvements in wide scale population testing, diagnosing all COVID cases globally remains an unreachable goal. A similar situation of underrecognizing and underreporting has also been proposed regarding long COVID cases [163]. Taking this argument into consideration, it can be safely assumed that the prevalence of sleep disturbances both during acute and during long COVID would be higher than reported, further augmenting the impact of the pandemic on public health.

## 10. Conclusions

Although essential for overall health, sleep seems to be partly neglected in the modern world. As such, sleep deprivation seems to accompany the modern lifestyle, affecting all age groups as a global phenomenon. While an extent of sleep deprivation to epidemic proportions can be debated before the current pandemic, it is clear that at least a significant part of the population experienced sleep deprivation and sleep disturbances.

Definitive conclusions are difficult to draw at this moment regarding the relationship between COVID-19 and sleep/mental health due to the marked heterogeneity of the available data and rapidly evolving knowledge.

The landscape created by the pandemic represented a challenge for society as a whole, with a significant effect on sleep and mental health in multiple population groups, such as the general population, health care workers and COVID-19 patients. Surprisingly, most reports seem to indicate an increase in sleep duration in the general population during the pandemic, but this change was mostly coupled with a decrease in sleep quality and altered sleep timing. Despite these changes, positive elements regarding sleep health have been observed in the younger segment of the population, mainly through reduced sleep debt and social jetlag, as determined by delayed school times. Sleep deficiencies are not among the most frequently reported symptoms of COVID-19. In spite of this fact, an increased prevalence of sleep deficiencies has been reported both in acute and long COVID patients. Overall decreases in mental health have also been reported, with anxiety, depression, stress and PTSD symptoms being the most frequently highlighted. Unsurprisingly, different population groups might not have been impacted in the same way by the pandemic, with most data seemingly pointing towards an increased burden on sleep and mental health in health care workers, as compared with the general population.

The relationship between COVID-19 and sleep/mental health can be seen as a bidirectional one. Consequently, the reported detrimental changes in sleep and mental health could further increase the burden of the pandemic by influencing the risk, severity and prognosis of the infection. Even tough data are not yet available, based on previous evidence, sleep deprivation and sleep deficiencies could also play a role in vaccine response.

## 11. Further Directions

Further research is needed to fully understand the short- and long-term effects of the current pandemic on sleep and mental health. As shown by the available evidence, the increased prevalence of these health problems has the potential to further increase the impact of the pandemic. As such, viable means of prevention that can be applied on a population level are warranted.

Additionally, the effect of sleep deprivation or sleep deficiencies on COVID vaccine efficiency also needs clarification in future studies.

## Figures and Tables

**Table 1:** Pre-pandemic prevalence of short sleep duration in adults.

Country	Datapoint	Prevalence (%) of Short Sleep	Reference
USA	1985	22	[26]
	1990s	13.7	[21]
	2004–2007	28.3	[25]
	2012	29.2	[26]
	2013	21	[27]
	2014	35.2 *	[31]
	2017	32.9	[29]
Canada	2013	7	[27]
Mexico	2013	11	[27]
The Netherlands, UK, USA	2000–2017	6.5 47 **	[28]
The Netherlands	2012	30.4	[30]
Britain	1990s	7.5	[21]
	Early 2000s	~13	[23]
UK	2013	18	[27]
Finland	Early 2000s	14.5	[22]
	1972–2005	~8–12 ^	[24]
Germany	2013	10	[27]
Japan	2013	19	[27]

* Short sleep duration of under 7 h as defined by the CDC (Centers for Disease Control and Prevention)—all other values refer to sleep duration of 6 h or less; ** Objective sleep data—unless specified otherwise, the presented values stem from self-reported sleep data; ^ According to age and gender.

**Table 2:** Pre-pandemic prevalence of short sleep duration in the younger segment of the population.

Country	Age Group	Datapoint	Prevalence of Short Sleep (%)	Definition of Insufficient Sleep or Sleep Duration	Reference
Multiple countries	Medical students	2001–2018	29	<6–8 h or NS	[32]
The Netherlands, UK, USA	14–17 years	2000–2017	51.5	<8–10 h	[28]
USA	High school students	2014	68.8 *	<8 h	[31]
	Middle school students High school students	2015	57.8 * 72.7 *	<8 h/<9 h ^	[33]
Canada	Secondary school students	2013–2016	49.7–54.7	<8–10 h	[34]
Norway	16–19 years	2012	53.8	<7 h	[35]
	16–17 years	2019	49.4 * 11.7 **	<7 h	[36]
Brazil	10–14 years	2014	12.6	<8 h	[37]
Saudi Arabia	10–19 years	2011–2012	45.6 * 33.4 **	<7 h	[38]

NS—Not specified; * Prevalence of short sleep duration on a school night; ** Prevalence of short sleep duration on free days/weekends; ^ According to age: <8 h for students aged 13–18 years and <9 h for students aged 6–12 years.

**Table 3:** Pre-pandemic prevalence of short sleep duration in the ageing segment of the population.

Country	Datapoint	Prevalence of Short Sleep (%)	Reference
USA	2014	26.3	[31]
Spain	2001	21.2	[42]
Poland	1980–1987	26.5	[44]
China	2005–2014	11.9	[41]
	1997–2016	26.7	[45]
Taiwan	1999–2002	53.9	[43]
	1993	14.6	[46]
Japan	2011–2013	21.6	[47]
Brazil	1997	17.7	[48]

Adults aged ≥60 years; short sleep duration of <6 h/≤6 h/<7 h.

**Table 4:** Comparison of the prevalence of sleep deficiencies * between the general population and health care workers during the pandemic.

Country	General Population (95% CI)		Health Care Workers (95% CI)		Reference
	Total Pooled Prevalence	PSQI Pooled Prevalence	Total Pooled Prevalence	PSQI Pooled Prevalence	
Multiple countries	**32.3%** (25.3–40.2)	**37.9%** (25.2–52.4)	**36%** (21.1–54.2)	**39.7%** (21.2–61.6)	[68]
Multiple countries	**18%** (15–21)	-	**31%** (27–36)	-	[72]
Multiple countries	-	-	**44%** (24.6–64.5)	-	[71]
China	-	-	**45.1%** (37.2–53.1)	**58%** (43.4–71.9)	[70]

PSQI—Pittsburgh Sleep Quality Index; * Sleep deficiencies as a broad term including sleep problems, sleep disturbance, sleep disorders.

**Table 5:** The persistence of at least one symptom during long COVID at different timeframes.

Timeframe	3 Months	1.5–6 Months	6 Months	8–10 Months	12 Months
Prevalence of at least one symptom	99.3% [118]	41% [119]	91% [120]	61% [121]	81% [122]
	-	-	76% [123]	-	-
	-	-	68% [124]	-	49% [124]
	-	-	61% [125]	-	-

**Table 6:** The prevalence of insomnia or sleep difficulties during long COVID at different timeframes.

Timeframe	1 Month	6 Months	8–10 Months	12 Months
Prevalence of insomnia or sleep difficulties	40% [127]	43% [126]	13.4% [121]	47% [122]
	-	38% [120]	-	-
	-	27% [124]	-	17% [124]
	-	26% [123]	-	-
	-	5% * [125]	-	-

* In a subset of home-isolated patients.

**Table 7:** Comparison of the prevalence of anxiety, depression and stress in the general population and health care workers during the pandemic.

Mental Health Symptoms	General Population	Health Care Workers
Anxiety	27.3–28.33% [74,138,139]	27.5–35.3% [71,74,138]
Depression	24.9, 26.7% [74,138]	27.05–35.4% [71,74,138]
Stress	51.7% [138]	56.5, 65.1% [71,138]

## Data Availability

All data are contained within the article.

## Abbreviations

ARI—acute respiratory infection; BMI—body mass index; CDC—Centers for Disease Control and Prevention; EEG—electroencephalogram; HCW—health care workers; ICU—intensive care unit; ILI—influenza-like illness; NIHR—National Institute for Health Research; NREM (sleep)—non-rapid eye movement sleep; OSA—obstructive sleep apnoea; PSG—polysomnography; PSQI—Pittsburgh Sleep Quality Index; PTSD—posttraumatic stress disorder; REM (sleep)—rapid eye movement sleep; RCSQ—Richards–Campbell Sleep Questionnaire.
